# Metformin-Associated Lactic Acidosis in a Diabetic Patient with Normal Kidney Function and Occult Cirrhosis

**DOI:** 10.1155/2022/5506744

**Published:** 2022-10-05

**Authors:** Jad Chidiac, Rebecca Kassab, Mirella Iskandar, Sahar Koubar, Mabel Aoun

**Affiliations:** ^1^Holy Spirit University of Kaslik, Lebanon; ^2^Faculty of Medicine, Saint-Joseph University of Beirut, Lebanon; ^3^Intensive Care Unit, Saint-George Hospital, Ajaltoun, Lebanon; ^4^Saint-John Hospital, Lebanese American University, Lebanon; ^5^Nephrology Department, University of Minnesota, Minneapolis, USA

## Abstract

**Background:**

Lactic acidosis is a well-known complication of metformin accumulation in diabetic patients with kidney failure. However, it is not usual to raise the diagnosis of metformin-associated lactic acidosis when patients have normal kidney function. The causes of metformin-induced high lactate include the accumulation of normal doses of metformin in chronic kidney disease, an overdose of this drug without kidney failure, or an increase in lactate production due to the inhibition of liver gluconeogenesis. *Case Presentation*. We report the case of a 61-year-old diabetic man who was brought to the emergency room in a comatose state. His family reported abdominal pain with diarrhea in the last two days. He was found to have severe lactic acidosis with normal serum creatinine. He was on a regular dose of metformin, and his family denied any other medical history or any alcohol abuse. He showed no signs of infection, his liver enzymes were slightly elevated, and he had severe anemia. His hemodynamics deteriorated quickly within hours, and an abdominal computed tomography scan revealed no abnormalities. He underwent a laparotomy that ruled out mesenteric ischemia and revealed an abnormal liver. The liver biopsy later confirmed the diagnosis of cirrhosis.

**Conclusions:**

We discuss here the probable causes of severe lactic acidosis and the role of metformin in exacerbating this acid-base disturbance in cirrhotic patients. Future research is needed to determine whether these patients might benefit from dialysis.

## 1. Background

Lactic acid, an end product of pyruvate under anaerobic conditions, is normally cleared by the liver and to a lesser extent by the kidneys [1]. Lactic acidosis occurs when lactic acid production exceeds its clearance and is generally defined as serum lactate levels that are above 4 mmol/L. It is characterized as being one of two types; type A is due to hypoperfusion that results in anaerobic glycolysis while type B involves causes unrelated to hypoperfusion, including liver disease, HIV, diabetes mellitus, and some medications such as metformin [2, 3]. Metformin is a first-line medication from the biguanide family used to treat type 2 diabetes, particularly in overweight individuals. It is widely used due to its low cost and its safety, as it rarely causes hypoglycemia [4, 5]. Its side effects include, but are not limited to, nausea, diarrhea, low blood sugar, and increased lactic acid levels. Because of the risk of lactic acidosis, metformin is used cautiously in conditions with high risk of anoxia or increase in lactate [6]. Metformin-associated lactic acidosis (MALA) is defined as lactate > 5 mmol/L, pH < 7.35, and concomitant exposure to metformin [7]. Ultimately, very high levels of lactic acid can lead to death through deleterious hemodynamic alterations [8]. Since the drug is cleared by the kidneys, it is wise to withhold its use in patients with advanced chronic kidney disease to avoid lactic acidosis. We discuss in this case report lactic acidosis secondary to metformin in the absence of kidney failure.

## 2. Case Presentation

### 2.1. Clinical History and Initial Laboratory Data

A 61-year-old Caucasian man was brought by ambulance to the emergency room for altered mental consciousness and hypotension. His medical history included smoking, hypertension treated with irbesartan and amlodipine, diabetes mellitus on metformin 850 mg bid, and vildagliptin/metformin 50 mg/1000 mg once a day. He was suffering from acute abdominal pain and worsening diarrhea in the last 48 hours, and he became hypotensive and comatose in the last hour.

On physical examination, his heart rate was 115 beats per minute, his blood pressure was 80/40 mmHg, oxygen saturation was 84%, and his body temperature was 36.7°C. His Glasgow score was 5. His abdomen was tender to palpation without a specific site of pain and slightly distended. No edema was seen on his lower extremities. His weight was estimated around 80 kg with a height of 172 cm.

Laboratory tests on admission showed leukocytosis, severe anemia, serum bicarbonate of 4 meq/L, a pH of 6.71, a serum albumin of 2.0 g/dL, a glycemia of 220 mg/dL, no ketonuria, elevated liver enzymes (2.5 times the normal value), and a serum creatinine of 0.9 mg/dL. His lactic acid was 284 mg/dL (normal range 4.5-19.8 mg/dl). Full details of his blood tests can be found in Table 1. The last available blood test goes back to six years ago and included a serum creatinine of 0.6 mg/dL and normal liver enzymes.

He was intubated and admitted to the intensive care unit. He received 5 liters of isotonic saline, and he was put on vasopressors to maintain his mean arterial pressure around 65 mmHg. His urine output was 150 mL per hour for the first three hours then dropped to 50 mL/hour until he developed anuria in the last few hours before death. He received within 12 hours 1220 meq of bicarbonate sodium 8.4% but his acidosis was resistant to treatment, and the highest pH level reached for a short time 7.05.

### 2.2. Additional Investigations and Treatment

**Figure 1:**
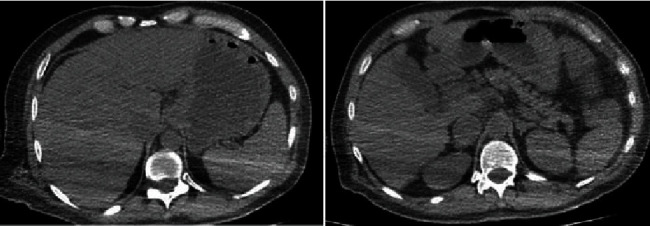
Abdomen CT scan showing a normal liver.

A total body computed tomography (CT) scan was performed and showed no significant abnormalities aside from mild bilateral pleural effusion and mild bowel distention but no kidney and liver abnormalities or ascites were noticed (Figure 1). The echocardiography showed a normal ejection fraction and no valvular disease. The nasogastric tube did not show signs of bleeding. The patient's condition continued to deteriorate, and broad-spectrum antibiotics were initiated. He was transfused with four blood units, and he received intravenous vitamin B1, B6, B12 and albumin. Blood cultures as well as urine culture were negative.

### 2.3. Diagnosis

Dialysis was discussed because of the possible metformin accumulation but the patient had normal urine output and no kidney failure. The patient's condition kept deteriorating, and he underwent an urgent laparotomy to rule out mesenteric ischemia. The surgeon found an abnormal liver and performed a liver biopsy. After a thorough investigation with the patient's family, it was found that he drank more alcohol than what was originally mentioned during the interrogation in the emergency room. The latest arterial blood gas after surgery showed a pH of 5.9 and a serum bicarbonate level of 5 mmol/L. The patient died 12 hours after his admission to the intensive care unit due to severe metabolic acidosis. A few days later, liver pathology results came back as micro- and macronodular cirrhosis of undetermined origin due to its advanced state.

## 3. Discussion and Conclusions

This case describes an unusual presentation of metformin-associated lactic acidosis (MALA) in a patient with unknown cirrhosis. The patient had severe acidemia, metabolic acidosis with a high anion gap of 33 mmol/L. The causes of high anion gap metabolic acidosis include intoxication with ethylene glycol or methanol, renal failure, salicylate intoxication, ketoacidosis (diabetic, alcoholic or starvation ketosis), or lactic acidosis. D-Lactate is found in very few cases and is produced by bacteria in case of short bowel syndrome [2]. The lactate that is usually measured is L-lactate, the main isomer in humans [2]. The very high level of L-lactate in our case confirmed the diagnosis of lactic acidosis and could result from a combination of high production and low elimination of lactate. Lactate is generated from pyruvate by the lactate dehydrogenase [1]. This lactate is produced mostly in muscles then cleared by the liver through the gluconeogenesis pathway, the respiratory chain, and the oxidation of pyruvate [1]. Tissue hypoxia induces lactate accumulation, and it is known to be the most common cause of lactic acidosis [1, 2]. Hypoperfusion secondary to septic or hemorrhagic shock or mesenteric ischemia were the most plausible hypotheses when the patient was first admitted [2]. First, septicemia was considered less likely with the low CRP, absence of fever, and sterile cultures. Despite this, the patient received broad-spectrum antibiotics. Second, hypoperfusion secondary to hypovolemia and hemorrhagic shock was possible given the low hemoglobin and macrocytosis but the nasogastric tube and the CT scan did not show any bleeding. The anemia of this patient was not due to hemolysis, given the normal haptoglobin, LDH, and reticulocytes. It remains unknown whether this patient had bleeding esophageal varices especially that he had an elevated serum urea level. It could be as well a chronic anemia of nutritional deficiency but the first workup did not include ferritin, vitamin B12, or folate measurement. The third reason of high lactate in a patient with abdominal pain is mesenteric ischemia. Although the CT scan did not confirm this diagnosis, the surgeon chose to perform a laparotomy that ruled out bowel ischemia but revealed an abnormal liver.

In addition to hypoperfusion, several other factors were needed to be ruled out when assessing this case of severe lactic acidosis. In a patient who might be alcoholic, thiamine deficiency that is a cofactor for pyruvate dehydrogenase could cause lactic acidosis and should be corrected [2]. Metformin as well may reduce thiamine absorption leading to reduced thiamine levels [9]. This could be another mechanism for the lactic acidosis observed with metformin [9]. In addition, metformin use inhibits liver gluconeogenesis and causes mitochondrial impairment increasing the risk of elevated lactate [2]. In patients on metformin who present with lactic acidosis, the physician searches immediately for a possible kidney failure. In kidney failure, lactic acidosis is caused by the accumulation of metformin, since it is primarily excreted in the urine. Caution is advised when using this drug in patients with chronic kidney disease and an estimated glomerular filtration rate (eGFR) < 30 mL/min^4^. To the best of our knowledge, there are no previous cases reported in the literature of MALA and normal eGFR levels. The hypothesis of metformin intoxication was promptly raised in our patient but regarded as very unlikely due to the normal renal function reflected by a normal serum creatinine level of 0.96 mg/dl and an eGFR of 90 mL/min at presentation. A prerenal acute kidney injury secondary to diarrhea and hypovolemia was possible in this case because of the lower serum creatinine six years ago. However, it was still very difficult to discuss metformin accumulation with that level of creatinine on admission and a normal urine output. Metformin is rapidly eliminated unchanged in urine, quicker than creatinine clearance [8]. Metformin has an estimated half-life of 2 to 6 hours, and it has been shown that 90% of this drug is eliminated in 12 hours [4, 7, 8]. Therefore, anyone with a normal urine output and kidney function is able to eliminate metformin except for cases of intoxication with very high doses of this drug. High doses of metformin are defined as >5 g/day in adults [7]. The latter was not the case in our patient.

**Figure 2:**
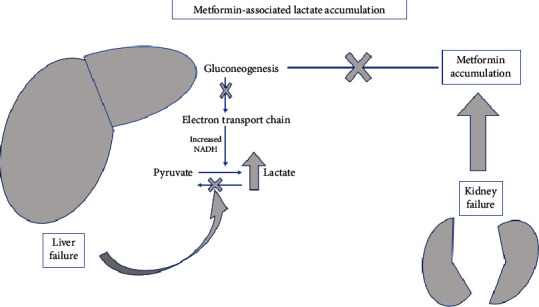
Metformin and lactic acidosis in liver or kidney failure.

The cause of lactic acidosis that was not discussed until later and that seems most probable in this patient is the abnormal lactate metabolism secondary to late-stage liver disease, exacerbated by metformin intake. The patient's state of shock probably resulted from severe acidemia as MALA itself can induce hypotension due to the reduction in systemic vascular resistance [10]. His blood tests showed mildly elevated liver enzymes, total bilirubin at upper limit of normal with a low Prothrombin Time and albumin. These values suggest an underlying liver disease but in the absence of any history of alcoholism or hepatitis, the diagnosis of liver cirrhosis was missed in the first hours. The patient did not develop hypoglycemia and his glucose levels were controlled without any treatment. The abdominal CT scan showed a normal liver but later, the liver biopsy confirmed the diagnosis of cirrhosis. A study from Italy in 2017 reported that liver disease was undiagnosed or unrecognized in two thirds of cases [11]. Another paper from Canada found that occult cirrhosis was frequent and represented 37% of cases of cirrhosis [12]. Moreover, the diagnosis of alcohol-related liver disease is usually delayed as showed by Subhani et al. in a recent paper from the UK where 47% of deaths secondary to alcohol-related liver disease received their first diagnosis in emergency hospital admissions [13]. In Lebanon, a single-center study in 2020 showed that alcohol-related liver disease represented 7.7% of all causes of chronic liver disease [14]. Liver disease could contribute to the accumulation of certain drugs and toxins. Lactic acid is oxidized in the liver back to pyruvate and later converted to glucose (Figure 2). If a patient has hepatitis or cirrhosis, the lactate metabolism is abnormal and will lead to a buildup of lactic acid in the body over time, then to serious metabolic acidosis and death. Gao et al. showed that 52% of critically ill patients with cirrhosis had metabolic acidosis and the mortality rate exceeded 40% [15]. In addition, a recent large study showed that metformin use in patients with cirrhosis is asscoiated with increased moratlity [16].

What remains controversial is whether a patient with cirrhosis and lactic acidosis would benefit from dialysis in the absence of kidney failure. The treatment of metformin accumulation in case of kidney failure includes heavy alkalinization, volume repletion, and dialysis. Based on the Extracorporeal Treatments In Poisoning (EXTRIP) workgroup, hemodialysis is recommended (1D, strongly recommended, with very low level of evidence) in cases of severe lactic acidosis, lactate concentration > 20 mmol/L, pH < 7.0, and failure of standard therapy (supportive care and bicarbonate) with impaired kidney function [7]. In a study of 117 MALA patients with acute kidney injury, the survival rate was as high as 78.3% following renal replacement therapy [17, 18]; however, the mean serum creatinine of these patents was 598.8 *μ*mol/l at the start of renal replacement therapy, which was not the case in our patient.

In cases of liver failure, the level of evidence for hemodialysis is still very weak (2D) and when hemodialysis is attempted, one should keep in mind that lactate handling and removal is very challenging in these cases [7]. With normal kidney function and adequate urine output, dialysis was not promptly initiated in our patient. He received major doses of bicarbonate but he did not improve. Based on the current evidence, hemodialysis does not decrease mortality in patients with severe shock and MALA [10]. We conclude that there is lack of evidence whether hemodialysis is beneficial in patients with shock, MALA, liver cirrhosis, and normal kidney function.

In conclusion, this case is a call to search for liver disease anytime a patient on metformin presents with severe lactic acidosis and normal kidney function. Without strong evidence, it remains unclear whether the patient can benefit from hemodialysis in the absence of kidney failure. These cases need to be published to build better evidence about this topic.

## Figures and Tables

**Table 1:** Laboratory results.

Test	T0 on admission	T9 presurgery	T15 postsurgery
Hemoglobin, g/dL	6.4	8.4	7.1
Hematocrit	21%	26%	22%
MCV	100	94	93
White blood cells	36,860	24,700	16,040
Platelets	250,000	155,000	117,000
Urea, mg/dL (mmol/L)	130 (7.15)	113 (6.21)	106 (5.83)
Creatinine, mg/dL (*μ*mol/L)	0.96 (84.88)	0.99 (87.54)	1.07 (94.61)
Sodium, mmol/L	127	143	148
Potassium, mmol/L	4.4	3.6	3.7
Bicarbonate	4	7	56
Chloride	90	97	96
pH (ABG)	6.71	7.01	6.98
pCO2, mmHg	24	23	23
Plasma osmolarity	300	—	—
Prothrombin time	46%	40%	—
INR	1.55	1.73	—
CRP, mg/dL (mg/L)	2.1 (21)	1.7 (17)	—
L-Lactate (normal: 4.5-19.8 mg/dl)	284 mg/dL or 31.5 mmol/L		
Reticulocytes' count	50000	—	—
LDH, U/L	430	—	—
Haptoglobin	0.43		
Protein, g/L	49	—	—
Albumin, g/L	20	—	—
Calcium, mg/dL (mmol/L)	8.8 (0.484)	9.8 (0.539)	8.7 (0.478)
Phosphorus, mg/dL (mmol/L)	9.2 (0.506)	9.1 (0.5)	8.7 (0.478)
Magnesium, mg/dL (mmol/L)	2.4 (0.132)	2.3 (0.126)	2.3 (0.126)
ASAT, U/L	103	—	—
GGT, U/L	95	—	—
Total bilirubin, mg/dL (mmol/L)	1.0 (0.055)	—	—
Direct bilirubin, mg/dL (mmol/L)	0.4 (0.022)	—	—
Lipase, U/L	81	—	—
Fibrinogen, mg/dL (*μ*mol/L)	232 (2.72)	—	—
D-Dimer, *μ*g/mL	0.1	—	—

Note: ABG: arterial blood gas; CRP: C-reactive protein; AST: aspartate aminotransferase; GGT: gamma-glutamyl transferase. T reflects time in hours since the admission to the emergency room; SI represents units in parenthesis.

## Data Availability

All data generated or analyzed during this study are included in this published article.

## Abbreviations

MALA: Metformin-associated lactic acidosis
CT: Computed tomography
ABG: Arterial blood gas
CRP: C-reactive protein
AST: Aspartate aminotransferase
GGT: Gamma-glutamyl transferase
eGFR: Estimated glomerular filtration rate
EXTRIP: Extracorporeal Treatments In Poisoning.
